# STIL, a peculiar molecule from styles, specifically dephosphorylates the pollen receptor kinase LePRK2 and stimulates pollen tube growth *in vitro*

**DOI:** 10.1186/1471-2229-10-33

**Published:** 2010-02-22

**Authors:** Diego L Wengier, María A Mazzella, Tamara M Salem, Sheila McCormick, Jorge P Muschietti

**Affiliations:** 1 Instituto de Ingeniería Genética y Biología Molecular (INGEBI), CONICET, Vuelta de Obligado 2490, 1428 Buenos Aires, Argentina; 2 Departamento de Fisiología y Biología Molecular y Celular, Facultad de Ciencias Exactas y Naturales, Universidad de Buenos Aires, Buenos Aires, Argentina; 3 Plant Gene Expression Center, United States Department of Agriculture/Agricultural Research Service, 800 Buchanan Street, Albany, California 94710, USA; 4 Department of Plant and Microbial Biology, University of California at Berkeley, Berkeley, California 94720, USA

## Abstract

**Background:**

LePRK1 and LePRK2 are two pollen receptor kinases localized to the plasma membrane, where they are present in a high molecular weight complex (LePRK complex). LePRK2 is phosphorylated in mature and germinated pollen, but is dephosphorylated when pollen membranes are incubated with tomato or tobacco style extracts.

**Results:**

Here we show that LePRK2 dephosphorylation is mediated by a heat-, acid-, base-, DTT- and protease-resistant component from tobacco styles. Using LePRK2 phosphorylation as a tracking assay for purification, style exudates were subjected to chloroform extraction, anionic exchange, and C18 reverse-phase chromatography columns. We finally obtained a single ~3,550 Da compound (as determined by UV-MALDI-TOF MS) that we named STIL (for **St**yle **I**nteractor for **L**ePRKs). STIL increased pollen tube lengths of *in vitro *germinated pollen in a dose-dependent manner.

**Conclusion:**

We propose that the LePRK complex perceives STIL, resulting in LePRK2 dephosphorylation and an increase in pollen tube growth.

## Background

In plants, pollination and subsequent fertilization rely on an extensive and complex dialog between the tissues of the pistil (both sporophytic and gametophytic) and the pollen tube [1,2]. Numerous proteins and other molecules from both the female and male are thought to regulate the biochemical dialog established when the pollen grain lands on the stigma, during pollen tube growth through the style and upon arrival at a synergid cell where the sperm cells are discharged. Some observations suggest that there is a hierarchy of signals in pollen tube germination and growth, wherein a pollen tube is unable to respond to late signals coming from the female gametophyte if it has not been previously exposed to early signals coming from the sporophyte [3]. This implies that pollen tubes have the ability to determine their geographical position within the female tissues and modify their physiology accordingly.

LePRK1 and LePRK2 are two LRR-receptor like kinases specifically expressed in pollen grains and tubes in *Solanum lycopersicum *(tomato) [4] and homologs of these proteins exist in other species [5]. These kinases localize to the plasma membrane and belong to a high molecular weight complex (LePRK) [6]; LePRK1 and LePRK2 bind different proteins from the pistil (such as LeSTIG [7]) or from pollen (LAT52 [8]; LeSHY [9]). LePRK2 is phosphorylated in mature and germinated pollen, but is specifically dephosphorylated upon incubation with style extracts [4]; this suggests that style components have the potential to regulate the LePRK complex biochemically [4]. We previously determined that this style component in tomato and tobacco had a molecular weight of 3-10 kDa and was heat-stable [6]. We also showed that LePRK1 and LePRK2 interact when expressed heterologously in yeast, and that this interaction can be dissociated by the addition of the same style fractions that promote LePRK2 dephosphorylation [6]. Recently Zhang et al. (2008) showed that antisense expression of LePRK2 resulted in pollen tubes with a reduced growth rate, suggesting that LePRK2 might be involved in pollen tube growth regulation [10]. A cytoplasmic protein called KPP [11], which is a ROPGEF [12], interacts with both LePRK1 and LePRK2. This interaction suggested a linkage between extracellular signals, receptor kinases, and modulation of ROP activities, which is critically important for pollen tube growth [13].

Numerous low molecular weight polypeptides have been implicated in signal transduction pathways in plants [14-16]. Some were isolated by biochemical purification, such as systemin ([17-19], phytosulfokine [20-22] and rapid-alkalinization factor (RALF) [23]. Specific physiological or biochemical effects were associated with these polypeptides [14,15] and their receptors were identified and biochemically characterized [systemins, [24-27]; phytosulfokines, [21,28-30]; RALF, [31], but early correlates of ligand binding, such as receptor de/phosphorylation, hetero-oligomerization or dissociation from interacting proteins, have not yet been shown. Other polypeptide ligands were first identified from mutant screens, such as CLAVATA3 (CLV3) [32] and TAPETUM DETERMINANT1 (TPD1) [33], by bioinformatics, such as the CLAVATA3/Embryo surrounding region-related peptides (CLE) [34,35], or by map-based cloning, such as the S-locus cysteine rich protein (SCR)/S-locus protein 11 (SP11) [36,37] among others [15]. Receptors for CLV3, TPD1 and SCR/SP11 have been identified [38-40]. Binding of SCR/SP11 to the S-locus Receptor Kinase (SRK) and TPD1 binding to the receptor kinase EXCESS MICROSPOROCYTES1 (EMS1) induce receptor autophosphorylation [39,40], and in the case of SCR/SP11-SRK, complex formation with the S-locus glycoprotein (SLG) [39].

In this paper, we describe the purification of STIL, a peculiar ~3,550 Da molecule from tobacco pistils that is responsible for LePRK2 dephosphorylation. STIL's activity is heat-, acid-, base-, DTT- and protease-resistant. Our results show that STIL promotes pollen growth from the onset of germination in a dose-dependent manner. We hypothesize that STIL's binding to the LePRK complex triggers LePRK2-specific dephosphorylation, which in turn modulates downstream components of the LePRK complex transduction pathways, such as ROPGEF [11,41] and probably ROP, resulting in pollen tube growth stimulation.

## Results

### STIL is a hydrophilic molecule that specifically promotes LePRK2 dephosphorylation

**Figure 1:**
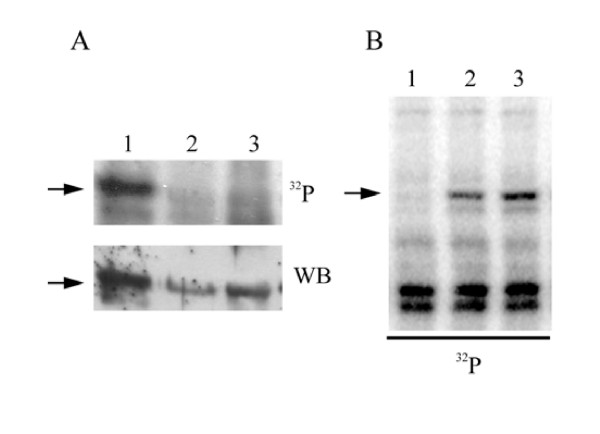
**Characterization of the LePRK2 dephosphorylating activity in tobacco style extracts**. **A**, Pollen microsomal fractions (15 μg) were incubated for 10 min with [gamma-^32^P]-ATP in buffer without (lane 1) or with (lane 2) tobacco style extract proteins (340 μg), or with (lane 3) tomato style exudate proteins (190 μg), then separated by SDS-PAGE, blotted onto nitrocellulose and subjected to autoradiography (top panel, ^32^P), then incubated with anti-LePRK2 antibody (bottom panel, Western Blot). The position of LePRK2 is indicated by arrows. **B**, Dephosphorylation activity after chloroform extraction of style extracts. Lane 1, aqueous phase; lane 2, interface; and lane 3, organic phase. The position of LePRK2 is indicated by an arrow.

We previously showed that LePRK2 is phosphorylated in tomato pollen microsomes, but specifically dephosphorylated when tomato stigma/style extracts were added during or after a phosphorylation reaction [4]. This suggested the presence of a LePRK2 dephosphorylating activity in tomato styles. Subsequently, we demonstrated that a 3-10 kDa heat-resistant molecule present in tomato and tobacco stigma/style extracts or exudates was responsible for this dephosphorylation [6] (and see Fig. 1A). To purify this molecule, we used LePRK2 dephosphorylation as a tracking assay. This molecule was named STIL for **St**yle **I**nteractor for **L**ePRKs.

In order to design a purification protocol, the behavior of STIL under various extraction or precipitation protocols was first evaluated. Most proteins can be precipitated from organic solvents [42]. However, Fig. 1B shows that STIL dephosphorylation activity was in the aqueous phase of a methanol-chloroform extraction and not in the organic phase or at the interface. This suggested that STIL did not have exposed hydrophobic moieties that in organic solvents partition to the organic phase. In contrast to typical proteins, STIL phosphorylation activity could not be precipitated by trichloroacetic acid, even in the presence of a carrier protein such as bovine serum albumin (data not shown), implying that STIL is highly soluble in salting out-low pH conditions, maybe because of the presence of negatively-charged highly hydrophilic residues on its surface.

We then evaluated the interaction of STIL with anionic and cationic exchange resin chromatography. The aqueous phase from methanol-chloroform-extracted stigma/style exudates was dried, dissolved in water and loaded onto solid-phase extraction cartridges. The resin was washed extensively with water and successively eluted with an ammonium bicarbonate gradient ending at 0.25 M. STIL dephosphorylation activity was retained and selectively eluted from an anionic exchanger, but not from a cationic exchanger (data not shown).

**Figure 2:**
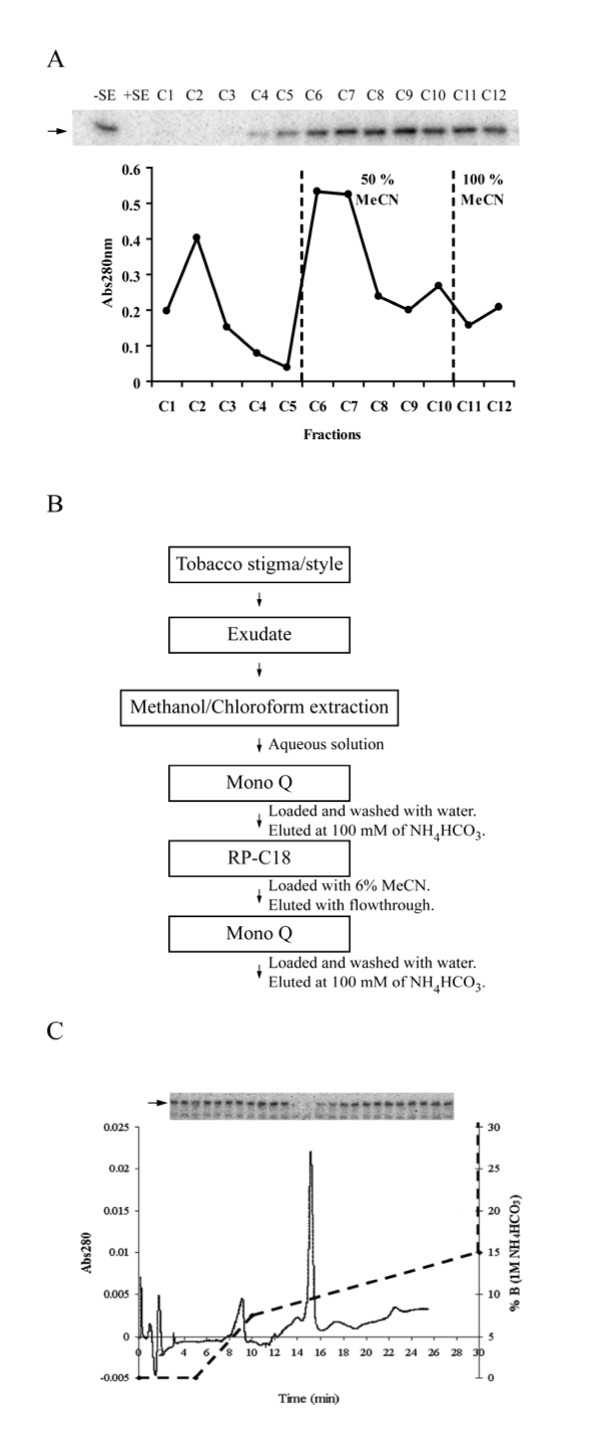
**Purification of STIL**. **A**, Chromatograph of the C18 reverse-phase semi-preparative column. The sample was loaded with 6% acetonitrile (MeCN) and eluted with 50% and 100% acetonitrile (indicated by dashed lines). Fractions were assayed for LePRK2 dephosphorylation activity (inset). The position of LePRK2 is indicated by an arrow. **B**, Purification protocol used for the isolation of STIL from tobacco stigma/style exudates. **C**, Chromatograph of the second Mono Q column. Abs280 nm (left vertical axis, solid line) and % of Buffer (100% Buffer corresponding to 1 M NH_4_HCO_3_; right vertical axis, dashed line). Fractions 3 to 27 were assayed for LePRK2 dephosphorylation activity (inset).

Peptides are often purified using C18 reverse-phase, solid phase extraction cartridges ([18,23], among others). Therefore, acetonitrile was added to a final concentration of 6% to an anionic exchanger-purified sample of STIL and loaded onto a C18 reverse-phase cartridge. The cartridge was washed with 6% acetonitrile until the absorbance at 280 nm reached basal levels and then was eluted with 50% and 100% acetonitrile, resulting in the elution of retained compounds (Fig. 2A). Surprisingly, STIL dephosphorylating activity was present in the flowthrough (6% acetonitrile fraction) but not in the eluates (inset of Fig. 2A). This result further supports the idea that STIL does not have exposed hydrophobic moieties, and thus does not interact with C18 reverse-phase resins.

### STIL purification protocol

Based on the behavior of STIL shown above, a purification protocol for STIL was developed (Fig. 2B). Although STIL was present in the flowthrough after C18 reverse-phase chromatography, this step was also included in order to purify STIL away from other 280 nm-absorbing compounds retained on this column (see Fig. 2A). Stigma/style exudates from tobacco pistils were extracted with methanol-chloroform and the aqueous phase was evaporated. The pellet was dissolved in water and loaded onto an anionic exchange column (MonoQ, GE Healthcare). Fractions that induced LePRK2 dephosphorylation were pooled together, concentrated and subjected to solid-phase extraction on a C18 reverse-phase cartridge. The sample was loaded in 6% acetonitrile and the flowthrough was collected and freeze-dried. The pellet was dissolved in water and loaded onto an anion exchange column (MonoQ). Fig. 2C shows that the fractions that dephosphorylated LePRK2 eluted as a 280 nm-absorbing single peak. As a result of the purification process, STIL was purified ~156,000 fold when compared to the starting exudate (Table 1). It is important to mention that the C18 solid-phase extraction step did not increase the purity of STIL more than the first anionic exchange column.

**Table 1:** Purification table.

Purification Step	**K2D**_**50**_	% Error	Purification Factor
Exudate	16.38	0.4	1.0
Chloroform supernatant	9.78	21.8	1.7
1st MonoQ*	7.35 × 10^-04^	22.9	22,276
C18*	1.28 × 10^-03^	18.6	12,769
2nd MonoQ	1.04 × 10^-04^	+	156,849

(*) No significant difference in a t-test (p > 0.05).

(+) No duplicates available.

K2D_50 _represents Abs280 units necessary for 50% dephosphorylation of LePRK2; values were obtained from an average of two independent purifications. % Error is calculated as (Standard error/K2D_50_) × 100. The purification factor was calculated by comparison to the starting exudate.

**Figure 3:**
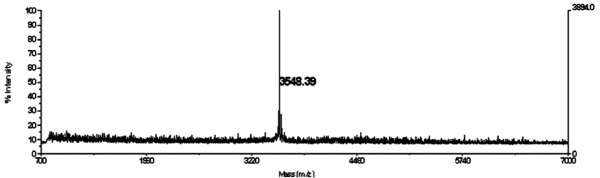
**UV-MALDI-TOF MS spectrum of pure STIL**. STIL is a 3,548.39 Da entity (spectrum corresponds to fractions 14 and 15, Fig. 2C).

**Figure 4:**
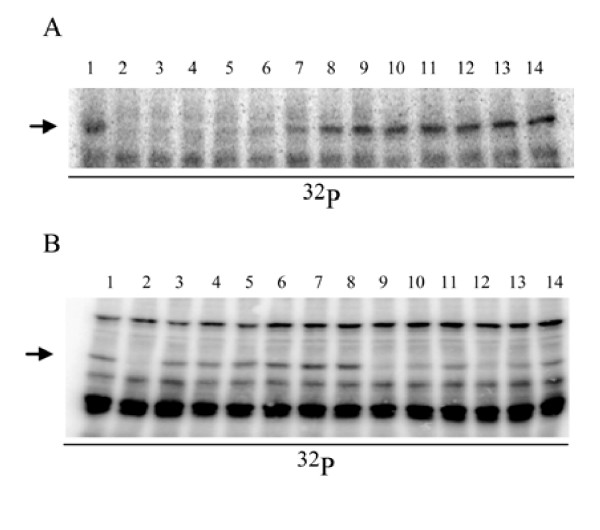
**STIL is labile to microwave-assisted acid hydrolysis**. **A**, Dilution series of the LePRK2 dephosphorylating activity of pure STIL. Lane 1, untreated pollen microsomal fraction. Lane 2 was treated with 4.8 × 10^-2 ^Abs280 units of tobacco style extract. Serial dilutions of purified STIL (lanes 3 through 14, from Fig. 2C) were used in the dephosphorylation assay; **B**, Pollen microsomal fractions were untreated (lane 1) or treated with tobacco style extract (lane 2), or with 1.44 Abs280 units (lanes 3, 6, 9 and 12), 0.72 Abs280 units (lanes 4, 7, 10 and 13) or 0.36 Abs280 units (lanes 5, 8, 11 and 14) of partially purified STIL (C18 percolate). Before the dephosphorylation assay was carried out, partially purified STIL (C18 percolate) was pre-treated by microwave-assisted acid hydrolysis (lanes 3-5). Lanes 6-8, salt concentration control. Lanes, 7-10, high temperature control. Lanes, 11-14, sample dilution control. The position of LePRK2 is indicated by an arrow.

The active fractions from the MonoQ column were pooled and subjected to UV-MALDI-TOF MS. Fig. 3 corresponds to one of 5 chromatographs from independent purifications where only one peak with a molecular mass of ~3,550 Da was consistently obtained; this result demonstrated that this entity corresponds to STIL and was purified to homogeneity. The fractions retained on the C18 reverse-phase cartridge (Fig. 2A) were used as controls for pollen germination assays (see below), and these were inactive (not shown) and never showed a peak with a molecular mass of ~3,550 Da in mass spectrometry determinations. Fig. 4A shows that when a dilution series of pure STIL was assayed in the LePRK2 dephosphorylation assay, 2.5 × 10^-3 ^absorbance units of STIL were able to dephosphorylate 50% of LePRK2 (lane 8). Moreover, the radioactive signal corresponding to phosphorylated LePRK2 was fully recovered only after diluting STIL to 4.8 × 10^-8 ^absorbance units (Fig. 4A, lane 14). These results indicate that dephosphorylation of LePRK2 was reduced when the amount of pure STIL was decreased.

### STIL is labile to microwave-assisted acid hydrolysis

To further biochemically characterize STIL, fractions of STIL were incubated with several proteases, such as trypsin, pepsin, carboxypeptidase Y, thermolysin and proteinase K, then the hydrolysates were tested for their ability to dephosphorylate LePRK2. None of the tested proteases inactivated STIL, since protease-treated STIL was still able to induce LePRK2 dephosphorylation (Table 2). The structural conformation of many low molecular weight peptides relies on numerous cysteine residues that stabilize them by disulfide bridges [43,44]. We tested the effect of incubating STIL at 100°C with 50 mM DTT for 15 min, which should disrupt disulfide bridges. The effects of base (1 N NaOH 100°C for 2 h) or acid (1 N HCl 100°C for 4 or 20 h) hydrolysis were also evaluated. Again, none of these treatments had any effect on the ability of STIL to dephosphorylate LePRK2 (Table 2). Therefore, a different and stronger acid hydrolysis protocol was evaluated. Microwave-assisted acid hydrolysis is commonly used for structural analysis of proteins and peptides [45-47]. LePRK2 could not be dephosphorylated after STIL was subjected to microwave-assisted acid hydrolysis, suggesting that this treatment totally or partially inactivated STIL (Fig. 4B).

**Table 2:** Biochemical characterization of STIL.

Treatment	LePRK2 dephosphorylation activity
1.5 N HCl (microwave)	-
1 N HCl 100°C 4 or 20 h	+
1 N NaOH 100°C 2 h	+
50 mM DTT 100°C 15 min	+
Pepsin	+
Carboxypeptidase Y	+
Trypsin	+
Proteinase K	+
Lyticase	+
Pectolyase	+

STIL was subjected to the listed treatments and the dephosphorylating activity was tested in a LePRK2 dephosphorylation assay (as described in Methods). STIL resistance to the treatment is indicated by "+", while "-" indicates STIL loss of activity.

Several pieces of evidence support that STIL is at least partially peptidic in nature: the presence of STIL always correlated with absorbance at 280 nm and amino acid determination (Table 3) showed that several amino acids are present in the STIL molecule. Amino acid determination relies on acid hydrolysis and derivatization. Since STIL activity is resistant to traditional acid hydrolysis, we expect that the full amino acid composition or the proportion of each amino acid has not been determined.

**Table 3:** Partial amino acid composition of STIL.

Amino acid	Detected amount (pmoles)
Serine	626
Alanine	542
Threonine	475
Lysine	443
Methionine	239
Arginine	173
Leucine	116

Pure STIL was subjected to 6 N HCl hydrolysis for 20 hours in a non-oxidizing environment, then derivatized and separated by gas chromatography. Amino acids were identified by comparing their retention times to standards.

### STIL promotes pollen tube growth from the onset of germination

**Figure 5:**
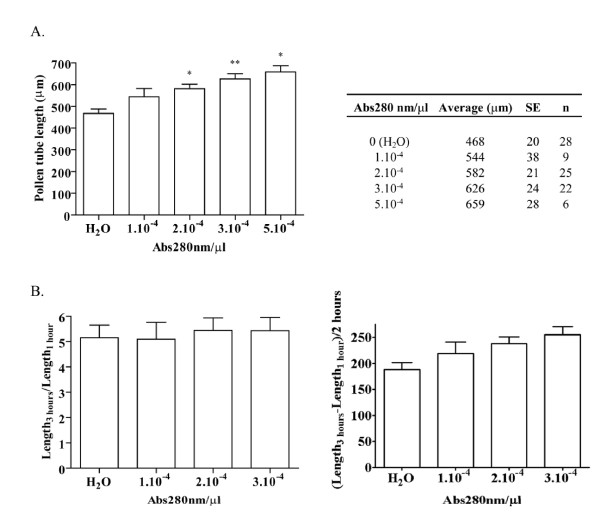
**STIL promotes *in vitro *pollen tube germination in tomato**. **A**, Mature pollen was germinated *in vitro *for 3 h in the presence of increasing amounts of partially purified STIL (C18 percolate). The concentration of STIL is expressed as Abs280 units/μl of Pollen Germination Medium. The Table summarizes the results. SE, standard error; n, number of replicates from independent experiments; *, significant differences relative to water control (*, p < 0.01; **, p < 0.001). **B**, Pollen tube length fold-increase (left panel) and growth rate (right panel) for different STIL concentrations. The standard error for each average (table in A) was used to calculate the error bars, using error propagation (partial derivatives).

LePRK2 has been implicated in transducing pistil signals to the pollen tube, resulting in the regulation of pollen tube growth [4,6,7,10]. This regulation is likely mediated by the regulation of ROP through KPP (a ROPGEF) [11]. Overexpression of a nearly full-length KPP in tomato or tobacco pollen resulted in the appearance of a swollen tip, a phenotype reminiscent of that seen when *At*ROP1 was overexpressed in tobacco pollen [48]. Furthermore, pollen tubes transiently over-expressing LePRK2 showed swollen tips [10]. These observations suggest that an extracellular signal such as STIL, which biochemically modulates the LePRK complex by LePRK2 dephosphorylation, could in turn affect pollen tube growth or/and morphology. Therefore the response of pollen tubes to increasing concentrations of purified STIL was evaluated, using *in vitro *germination assays. No aberrant phenotypes, such as swollen tips, were observed in any of the treatments. After 1 h of germination, only the highest concentration of STIL assayed (0.0003 Abs280 nm/μl of Pollen Germination Medium, PGM) resulted in a significant statistical (p < 0.05) increase in pollen tube length (115.20 ± 6.58, n = 9) when compared to pollen tube length in PGM in the absence of STIL (90.79 ± 4.86, n = 11). However, after 3 h of germination, pollen tubes in the presence of increasing concentrations of STIL were significantly longer than those germinated in the absence of STIL (Fig. 5A) or in the presence of fractions retained on the C18 reverse-phase cartridge (not shown; Fig. 2A see above). This stimulation of growth could be a response to the perception of STIL from the onset of germination or it could be achieved only after pollen tubes are capable of perceiving STIL. To discriminate between these two hypotheses, pollen tube length fold-increase and growth rate were calculated. If STIL is perceived from the onset of germination, then pollen tube growth rate would depend on STIL concentration and higher concentrations of STIL would result in longer pollen tubes but with constant fold-increases in pollen tube length. Alternatively, if the perception of STIL is delayed until tubes have formed, then pollen tube growth rate initially would be constant and, when pollen tubes are able to respond, rates would increase in response to STIL concentrations. This behavior would be reflected in larger fold-increases in pollen tube length, in response to higher concentrations of STIL. Fig. 5B shows that pollen tube growth rate increased depending on STIL concentration but that the fold-increase in pollen tube length showed no differences between treatments. These results suggest that STIL is perceived from the onset of germination.

## Discussion

STIL is a peculiar molecule and a potential extracellular partner for the tomato pollen LePRK complex. Preliminary biochemical characterization indicated that STIL is a negatively charged, hydrophilic compound that absorbs at 280 nm. From the amino acid determination, we can conclude that STIL is at least partially peptidic. However, since neither 280 nm-absorbing residues nor negatively charged amino acids were identified, the amino acid determination was partial, possibly because of the resistance of STIL to the standard acid hydrolysis conditions commonly used for amino acid determination. Some proteins are prone to aggregation when heated during acid hydrolysis, making them recalcitrant to degradation [45]. If STIL has a hydrophobic core with 280 nm-absorbing residues and a hydrophilic surface exposed to the medium, only superficial amino acids would be susceptible to acid hydrolysis. We tried several mass spectrometry approaches in order to determine the structure of STIL (not shown), but none were successful in determining STIL's full structure. The high mass of the molecular ion and its resistance to enzymatic fragmentation are major obstacles in determining STIL's structure. So far, UV-MALDI-TOF tandem mass spectrometry analysis confirmed the presence of a short tract of amino acid residues (R-R-S or R-S-R) in STIL (data not shown). Considering its molecular mass as determined by MALDI, STIL could be a peptide of ~30 amino acids.

We showed that STIL's biochemical activity is resistant to drastic treatments, such as incubations with acid or alkali under high temperatures, or DTT reduction, suggesting that STIL corresponds to a stable molecule and that STIL must have a peculiar structure in order to withstand those extreme conditions. Its resistance to several proteases, even though the target amino acids for these enzymes are present in STIL (Table 3), further supports this idea. It is possible that some of these treatments had an effect on the structure of STIL, but none (except microwave-assisted acid hydrolysis) affected its ability to dephosphorylate LePRK2. There are several explanatory hypotheses as to how microwave-assisted acid hydrolysis permits the breakage of peptidic bonds in polypeptides when traditional acid hydrolysis has failed [49], but it is not known if overheating of the sample (up to ~170-180°C) and high pressure, and/or abolition of protein aggregation causes efficient hydrolysis.

There are several reports of other low molecular weight peptides with partial resistance to extreme treatments. For example, bacterial endotoxins [50] are resistant to proteases and acid treatments, tick microfilins [51] and pig cerebroside sulfate activator [52] are heat stable and partially resistant to proteases. However, none of them share all the properties shown by STIL. Another example is cyclotides, which are circularized peptides found in the plant families Violaceae, Rubiaceae and Cucurbitaceae [43]. Cyclotides are heat stable and are resistant to proteases and to acid hydrolysis. Their N- and C- termini are covalently linked and three intramolecular disulfide bridges stabilize their three dimensional structure, resulting in an extremely compact molecule [43,53,54]. However, plant cyclotides are easily purified by reverse-phase purification because 40 to 50% of their primary structure corresponds to hydrophobic residues, whereas STIL was found in the flowthrough of a C18 column.

In this paper, we showed that STIL promotes pollen tube growth from the onset of germination. Several factors, such as lipids and proteins, are involved in pollen tube growth, guidance and adhesion. Lipids are thought to provide a directional cue to the developing pollen tubes by controlling the flow of water [55-57]. A ~9 kDa lily stigma/stylar cysteine-rich adhesin (SCA) with some sequence similarity to lipid transfer proteins was associated with pollen tube adhesion and was first described as an extracellular "glue" for pollen when associated with pectin [58-60]; SCA also participated in pollen tube guidance when acting together with chemocyanin, a blue copper protein of the plantacyanin family [61]. Nonetheless, no immediate biochemical response to SCA was found in growing pollen tubes, nor is there additional information for a signal transduction pathway involved in pollen tube reorientation by chemocyanin or its Arabidopsis homolog, plantacyanin [61,62]. Arabinogalactan proteins (AGPs) have also been involved in modulating pollen tube growth in Solanaceous species. A transmitting tissue-specific (TTS) AGP in tobacco acted as a signal directing pollen tube growth towards the ovary and was required for establishing normal growth rates [63,64], but there is no biochemical evidence for a signal transduction pathway involved in pollen tube growth stimulation or, specifically, in TTS-mediated pollen tube reorientation.

There are at least three potential ligands, i.e. LAT52, LeSHY and LeSTIG1 [7-9] for the LePRK complex. STIL is different from these proteins. LAT52 and LeSHY are pollen-expressed proteins of ~20 kDa and ~35 kDa, respectively. LeSTIG1 is a stigma-expressed protein of ~15 kDa, but it does not induce LePRK2 dephosphorylation (data not shown). Our results suggest that STIL might be another female partner for the LePRK complex.

LePRKs were first implicated in pollen tube growth signal transduction due to their mRNA expression during late pollen development and their protein localization [4]. A possible model is that the binding of extracellular cues from female tissues to the LePRK complex regulates KPP activity, leading to the activation of ROP and the modulation of pollen tube growth [11]. Zhang and McCormick [41] provided more support for the role receptor kinases play in modulating ROPGEF activity. In Arabidopsis, AtROPGEF12, a homolog of KPP, interacts via its C-terminus with the cytoplasmic domain of AtPRK2a, a homolog of LePRK2. C-terminal phosphorylation of AtROPGEF12 by AtPRK2a was proposed to release an intramolecular inhibition of AtROPGEF, leading to the promotion of pollen tube growth [41]; this implies that AtPRK2a (and maybe also LePRK2) has a major role in pollen tube growth modulation. Recent results support this hypothesis, since antisense expression of LePRK2 resulted in pollen tubes with reduced growth rate [10]. Furthermore, the growth stimulation of pollen tubes by STIL is completely dependent on the presence of LePRK2, since LePRK2 antisense plants are unresponsive to STIL [10].

## Conclusions

In our model, STIL action is associated with receptor dephosphorylation, which in turn would lead to the activation of proteins present in the LePRK complex and to pollen tube growth. The idea that STIL is a ligand is supported by the apoplastic localization of STIL, the immediate biochemical response to its presence (LePRK2 dephosphorylation) and that STIL stimulates pollen tube growth from the beginning of germination. These observations pose an interesting question about the paradigm of signaling transduction through receptor kinases in general, where binding of the ligand to the extracellular domain of a receptor leads to auto-phosphorylation of its cytoplasmic domain, aggregation with other plasma membrane proteins and transduction of the signal by phosphorylating downstream effectors [65-67]. In this context, determination of the molecular structure of STIL and demonstrating that it can bind to the LePRK complex will be essential to confirm that STIL is a bona fide ligand of the LePRK complex.

## Methods

### Plant Material

*Solanum lycopersicum *cv. VF36 and *Nicotiana tabacum *cv. Xanthi D8 plants were grown under standard greenhouse conditions. Tomato pollen was obtained by vibrating flowers, as described before [4]. Tomato or tobacco pistils were harvested from mature flowers, the ovaries cut away and the remaining stigma/styles stored at -80°C until future use.

### Pollen Protein Extraction

Fifty mg of mature pollen were disrupted in 0.5 ml of extraction buffer [50 mM Tris-HCl, pH 7.4; 1 mM EDTA; 50 mM NaCl; 1× protease inhibitor cocktail (Complete; Roche Molecular Biochemicals)] by grinding 5 times for 1 min in a 7 ml Tenbroeck glass grinder (Kontes). The homogenate was centrifuged at 4°C for 15 min at 10,000 g. The supernatant was centrifuged at 4°C for 1.5 h at 100,000 g and the pellet (P_100_) containing microsomal membranes was resuspended in extraction buffer supplemented with 0.5% Nonidet P-40, by stirring on a magnetic stirrer at 0°C for 1 h.

To obtain total protein extracts, mature pollen was disrupted using extraction buffer containing detergent (0.5% NP-40). The resulting homogenate was stirred on a magnetic stirrer at 4°C for 1 h and centrifuged at 4°C for 15 min at 10,000 g, and then the supernatant was fractionated by centrifugation at 4°C for 1.5 h at 100,000 g. The second supernatant (total protein extract) was stored at -80°C until further use.

### LePRK2 Dephosphorylation Assay

A phosphorylation stock was prepared with 1× phosphorylation buffer (50 mM HEPES; 2 mM MnCl_2_; 2 mM MgCl_2_; 1 mM CaCl_2_; 1 mM DTT) and 15 μg of pollen microsomal proteins per reaction. Every treated or untreated stigma/style sample to be tested for dephosphorylation capacity was diluted with water or buffer to a predetermined volume and 5× phosphorylation buffer was added to a final concentration of 1×. The phosphorylation reaction was started by completing the phosphorylation cocktail with 0.125 μCi of [gamma-^32^P]-ATP per reaction to the phosphorylation stock, mixing and delivering 6 μl of the cocktail to each sample (15 μg pollen microsomal protein + 0.125 μCi of [gamma-^32^P]-ATP in 1× phosphorylation buffer). The reaction was incubated at room temperature for 10 min and stopped by protein precipitation with trichloroacetic acid (5% final concentration). Samples were centrifuged at room temperature for 5 min at 10,000 g, then supernatants were discarded and pellets were resuspended with sample buffer (500 mM Tris-HCl pH 8; 2% SDS; 10% glycerol; 5% β-mercaptoethanol; 0.001% bromophenol blue). Samples were incubated at 100°C for 3 min, centrifuged at room temperature for 3 min at 10,000 g and proteins in the supernatant were separated by 8% SDS-PAGE. Gels were blotted to nitrocellulose and the radioactive signal was detected with a Storm 820 PhosphorImager (Molecular Dynamics).

For immunoblotting, membranes were blocked first with 4% nonfat dry milk and 2% glycine in Tris-buffered saline (TBS) with 0.2% Triton X-100 for 30 min at room temperature. The blocked membranes were incubated with antibodies against LePRK2 [4] diluted to 1:1000 in TBS with 0.2% Triton X-100, 2% nonfat dry milk, and 2% glycine for 1 h, with shaking at room temperature. After three washes of 10 min each with TBS with 0.2% Triton X-100, the membranes were incubated for 1 h at room temperature with sheep anti-mouse polyclonal secondary antibodies conjugated with horseradish peroxidase (GE Healthcare Life Sciences) diluted 1:5000 in TBS with 0.2% Triton X-100, 2% nonfat dry milk and 2% glycine. Afterwards, the membranes were washed and developed using an enhanced chemiluminescence kit (GE Healthcare Life Sciences).

### STIL methanol-chloroform extraction and microwave-assisted acid hydrolysis

For methanol-chloroform extraction, two volumes of methanol and one volume of chloroform were added to two volumes of stigma/style exudates, and then vigorously shaken. The extract was centrifuged at room temperature for 5 min at 10,000 g and the supernatant was transferred to a new tube. The protein interface was precipitated by adding 9 volumes of methanol to the interface and organic phase, mixing and centrifuging at room temperature for 5 min at 10,000 g. The second supernatant, corresponding to the organic phase, was transferred to a new tube. Samples corresponding to aqueous phase, interface and organic phase were dried to completion in a rotary evaporator and dissolved in water.

Microwave-assisted acid hydrolysis was performed according to Zhong *et al*. [45]. Hydrolysis was performed on a STIL-enriched fraction corresponding to the C18 percolate fraction that specifically dephosphorylated LePRK2. Hydrochloric acid was added to two volumes of STIL to a final concentration of 1.5 N in 50 μl. Three different controls were prepared: 1) heat control, STIL diluted to a final volume of 50 μl, omitting HCl, and heated; 2) salt control, 50 μl of 1.5 N HCl was heated, omitting STIL; and 3) dilution control, STIL was diluted as for the acid-treated sample (as mentioned above), but omitting HCl and heat. Microtubes were sealed with Parafilm, locked with cap locks and heated in a microwave oven in which a non-hermetic capped tray containing 100 ml of deionized water was also present, for 10 min at 900 W. After treatment, the pH of acid-containing samples and controls were equilibrated with NaOH and Tris-HCl, pH 8 (0.1 M, final concentration). NaCl was added to the heat control to a final concentration of 1.5 N. Finally, samples and controls were diluted to 100 μl and 25 (1.44 Abs280 units), 12.5 (0.72 Abs280 units) or 6.25 (0.36 Abs280 units) μl were assayed in the LePRK2 dephosphorylation assay.

### Amino acid determination

Amino acid determination is based on acid hydrolysis of the sample, derivatization and separation by gas chromatography [68]. Analysis was performed at the LANAIS-PRO-CONICET, Facultad de Farmacia y Bioquímica-University of Buenos Aires, Buenos Aires, Argentina, following standard procedures.

### STIL purification

Exudates were obtained by cutting 100 tobacco styles (including stigmas) transversely in 5 mm segments and incubating overnight in 25 ml of 50 mM ammonium bicarbonate at 4°C with gentle agitation. The exudate was filtered through miracloth and Whatman filter paper (grade No. 1) and then subjected to chloroform-methanol extraction. The aqueous phase was dried by rotary evaporation and the pellet was dissolved in MilliQ water. The dissolved pellet was centrifuged 10 min at 10,000 g and the supernatant was fractionated by FPLC on a Mono Q 5/50 GL Monobead™ column (GE Healthcare Life Sciences). Fractionation was performed at 1 ml/min and was started by loading the sample in water, followed by 5 min of water, then a 0 to 75 mM ammonium bicarbonate gradient over 5 min and a 75 to 100 mM ammonium gradient over 10 min. The presence of STIL was determined in every fraction by a LePRK2 dephosphorylation assay. Fractions that showed LePRK2 dephosphorylation were pooled, freeze-dried, dissolved in 6% acetonitrile and subjected to solid-phase extraction in a Sep-Pak™ Plus C18 cartridge (Waters). The cartridge was thoroughly washed with 6% acetonitrile and the percolate (corresponding to a highly enriched fraction of STIL) was collected until the absorbance at 280 nm dropped to basal levels. The percolate was freeze-dried in order to eliminate acetonitrile and this fraction was reloaded in a Mono Q 5/50 GL Monobead™ column and separated as mentioned before. Finally, fractions capable of dephosphorylating LePRK2, corresponding to pure STIL (as determined by UV-MALDI-TOF mass spectrometry; see Fig. 3), were desalted by repeatedly vacuum drying in a rotary evaporator. A 1/2-dilution series of STIL was assayed for LePRK2 dephosphorylation (Fig. 4A). Dilutions tested correspond to 0.0484, 0.0242, 0.01188, 0.00484, 0.002398, 0.000484, 0.0002398, 0.0001188, 0.0000484, 0.00002398, 0.00001188 and 0.00000484 Abs280 units.

### Germination of Pollen

Freshly collected pollen was prehydrated in Pollen Germination Medium [PGM, 24% polyethylene glycol 3350; 2% sucrose; 20 mM MES pH 6; 0.02% p/v MgSO_4_; 0.01% p/v KNO_3_; 0.01% p/v H_3_BO_3_; 0.07% p/v Ca(NO_3_)_2_] [4] but without sucrose for 30 min at room temperature with occasional gentle agitation. After incubation, the pollen suspension was centrifuged for 5 min at 3,000 g and resuspended to a final concentration of 1 mg pollen/ml of complete PGM without additives (H_2_O) or supplemented with 0.0001, 0.0002, 0.0003 or 0.0005 Abs280 units of STIL/μl of PGM. Every experiment included 3 or more replicates for each treatment. Pollen germination was carried out for 3 hours at 28°C and 50 rpm in an orbital shaker in 24-well microplates, each well containing 400 μl of the pollen suspension. After germination, the pollen suspension was transferred to 1.5 ml microtubes and 10× fixing solution (5.6% formaldehyde; 0.5% glutaraldehyde; 25% PEG 3350) was added to a final concentration of 1×. Samples were incubated 30 min at 4°C with gentle agitation. Fixed pollen tubes were observed with an inverted microscope Axiophot (Zeiss, Jena, Germany) and 50 pictures were taken for each replicate with a digital camera (Diagnostic Instruments, Sterling Heights, MI). Fifteen pictures were randomly selected and the lengths of all the pollen tubes in each picture were determined using AxioVision software (Zeiss) and averaged. Pollen tube lengths for each replicate were calculated as the average from all 15 values previously obtained. To compare the effects of STIL to control treatments, ANOVA was performed using Prism (version 4.03 for Windows; GraphPad Software Inc) after verification of normality and homogeneity of variances. Germination assays were repeated six times. Fold-increase in pollen tube length was calculated as L3h/L1h; and growth rate as (L3h-L1h)/2 h, where L3h corresponds to average pollen tube length after 3 hours of germination and L1h corresponds to average pollen tube length after 1 hour of germination, for a given STIL concentration.

## Authors' contributions

DLW carried out the biochemical and physiological studies, and wrote the manuscript. MAM was in charge of designing the physiological experiments, helped with the statistical analysis and helped write the manuscript. TMS helped by discussing the experimental design and helped write the manuscript. SM participated in the design of the experiments through critical discussion and helped write the manuscript. JPM conceived the study, participated in its design and coordination and wrote the manuscript. All authors read and approved the final manuscript.
